# Interrelationship Between the Morning-to-Evening Changes in Home Blood Pressure and Pulse Rate

**DOI:** 10.1093/ajh/hpaf137

**Published:** 2025-07-20

**Authors:** Jia-Bo Zhu, Qian-Hui Guo, Yi Zhou, Wen-Yuan-Yue Wang, Yuan-Yuan Kang, Xiao-Fei Ye, Xin-Yu Wang, Ming-Xuan Li, Yan Li, Ji-Guang Wang

**Affiliations:** Department of Cardiovascular Medicine, Centre for Epidemiological Studies and Clinical Trials, Shanghai Key Laboratory of Hypertension, The Shanghai Institute of Hypertension, National Research Centre for Translational Medicine at Shanghai, Ruijin Hospital, Shanghai Jiao Tong University School of Medicine, Shanghai, China; Department of Cardiovascular Medicine, Centre for Epidemiological Studies and Clinical Trials, Shanghai Key Laboratory of Hypertension, The Shanghai Institute of Hypertension, National Research Centre for Translational Medicine at Shanghai, Ruijin Hospital, Shanghai Jiao Tong University School of Medicine, Shanghai, China; Department of Cardiovascular Medicine, Centre for Epidemiological Studies and Clinical Trials, Shanghai Key Laboratory of Hypertension, The Shanghai Institute of Hypertension, National Research Centre for Translational Medicine at Shanghai, Ruijin Hospital, Shanghai Jiao Tong University School of Medicine, Shanghai, China; School of Public Health, Shanghai Jiao Tong University School of Medicine, Shanghai, China; Department of Cardiovascular Medicine, Centre for Epidemiological Studies and Clinical Trials, Shanghai Key Laboratory of Hypertension, The Shanghai Institute of Hypertension, National Research Centre for Translational Medicine at Shanghai, Ruijin Hospital, Shanghai Jiao Tong University School of Medicine, Shanghai, China; School of Public Health, Shanghai Jiao Tong University School of Medicine, Shanghai, China; Department of Cardiovascular Medicine, Centre for Epidemiological Studies and Clinical Trials, Shanghai Key Laboratory of Hypertension, The Shanghai Institute of Hypertension, National Research Centre for Translational Medicine at Shanghai, Ruijin Hospital, Shanghai Jiao Tong University School of Medicine, Shanghai, China; Department of Cardiovascular Medicine, Centre for Epidemiological Studies and Clinical Trials, Shanghai Key Laboratory of Hypertension, The Shanghai Institute of Hypertension, National Research Centre for Translational Medicine at Shanghai, Ruijin Hospital, Shanghai Jiao Tong University School of Medicine, Shanghai, China; Department of Cardiovascular Medicine, Centre for Epidemiological Studies and Clinical Trials, Shanghai Key Laboratory of Hypertension, The Shanghai Institute of Hypertension, National Research Centre for Translational Medicine at Shanghai, Ruijin Hospital, Shanghai Jiao Tong University School of Medicine, Shanghai, China; Department of Cardiovascular Medicine, Centre for Epidemiological Studies and Clinical Trials, Shanghai Key Laboratory of Hypertension, The Shanghai Institute of Hypertension, National Research Centre for Translational Medicine at Shanghai, Ruijin Hospital, Shanghai Jiao Tong University School of Medicine, Shanghai, China; School of Public Health, Shanghai Jiao Tong University School of Medicine, Shanghai, China

**Keywords:** blood pressure, home blood pressure monitoring, hypertension, pulse rate, morning, evening

## Abstract

**BACKGROUND:**

We investigated the morning-to-evening changes in home blood pressure (BP) and pulse rate for demographic and clinical determinants, interrelationship, and association with BP control in treated patients with hypertension.

**METHODS:**

We performed a cross-sectional analysis in patients (≥55 years of age) with hypertension, enrolled in a China nationwide registry on home BP monitoring between 2020 and 2025. Home BP was measured three times consecutively in the morning and evening, respectively, for seven consecutive days. The change was calculated by subtracting the BP and pulse rate values in the morning from those in the evening.

**RESULTS:**

The 4,787 participants had a mean (±SD) age of 66.1 (±7.5) years, and included 2,366 (49.4%) men. Overall, systolic/diastolic BP decreased from 129.1/80.6 mmHg in the morning to 127.2/78.7 mmHg in the evening by a mean change of −1.9 ± 7.8/−1.8 ± 4.7 mmHg. Pulse rate, however, increased from 70.5 beats/min in the morning to 73.7 beats/min in the evening by a mean change of +3.1 ± 5.8 beats/min. Adjusted analyses showed that the changes in pulse rate were negatively associated with those in both systolic (*r* = −0.20, 95% CI: −0.22 to −0.17) and diastolic BP (*r* = −0.12, 95% CI: −0.14 to −0.09). Patients with a change in pulse rate above the median (≥3.0 beats/min) had a lower control rate of office systolic/diastolic BP (60.1% vs. 65.5%, *P* < 0.001) than those with a change in pulse rate below the median.

**CONCLUSIONS:**

There were interrelated morning-to-evening changes in home BP and pulse rate, being a drop and rise, respectively.

## Graphical Abstract

**Graphical Abstract:**
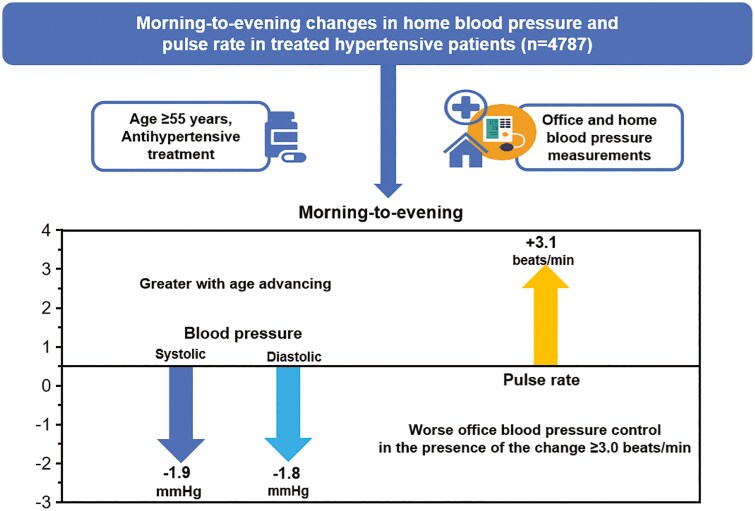

Current hypertension guidelines recommend the use of home blood pressure monitoring (HBPM) for the management of hypertension.^1–4^ As one of the out-of-office blood pressure measurement techniques, HBPM may improve prognostic assessment for hypertension-mediated organ damage and cardiovascular clinical outcomes. In addition, it is complementary to ambulatory blood pressure monitoring in the differentiation of white-coat from sustained hypertension and identification of masked hypertension from true normotension.^5–9^

HBPM usually requires 2–3 blood pressure measurements in the morning and evening for seven consecutive days. The evening measurements can be performed either before or after supper, depending on the time of supper. Regardless of whether the evening measurements are performed before supper, such as in Europe or United States,^1,2,10^ or after super in Asia,^3,4,11,12^ there is a morning-to-evening blood pressure difference. There is evidence that a big difference, for instance, greater than 20 mmHg, is associated with unfavorable clinical outcomes.^13–15^ However, few studies have ever investigated the determinants of the morning-to-evening blood pressure difference, especially with regard to its relationship with the possible changes in pulse rate. In the present study, we investigated the morning-to-evening changes in home blood pressure and pulse rate, and their association with office blood pressure control.

## METHODS

### Study Population

We performed cross-sectional analysis in treated patients (≥55 years of age) with hypertension, enrolled in an ongoing China nationwide registry on HBPM (Action of Controlling Home Blood Pressure to Target in Ten Thousand Patients). For inclusion, outpatients seen in a hospital or a community health center had to be ≥55 years, and had been treated with antihypertensive drugs. We excluded hospitalized patients and those newly diagnosed and untreated hypertensive patients. The study protocol was approved by the ethics committee of Ruijin Hospital, Shanghai Jiaotong University School of Medicine, Shanghai, China. All study participants gave informed written consent.

### Office and Home Blood Pressure Measurements

Office blood pressure was measured three times consecutively with a 1-minute interval using a validated, automated upper arm cuff device (HEM-9200T Omron Healthcare, Kyoto, Japan),^16^ after resting for at least 5 minutes in the sitting position. An appropriately sized cuff was used. These three readings of blood pressure and pulse rate were averaged for analysis.

All participants were instructed for standardized HBPM,^4^ especially for placing an appropriately sized cuff around his or her non-dominant arm. HBPM was performed using the same model of automated blood pressure monitor (Omron HEM-9200T) for seven consecutive days. Blood pressure was measured three times consecutively with a 1-minute interval after resting for at least 5 minutes in the sitting position. Blood pressure in the morning was measured within 1 hour of waking up, before drug intake and breakfast, and after urination, without vigorous exercise. Blood pressure in the evening was measured after super and a shower or bath and before going to sleep.^4^ Blood pressure and pulse rate readings were automatically transmitted to a digital platform for storage, analysis, and reporting. All home blood pressure and pulse rate readings were averaged for analysis.

### Questionnaire and Anthropometric and Biochemical Measurements

A standardized questionnaire was administered to collect information on medical history, lifestyle, and use of medications. Body weight was measured with light indoor clothing and without shoes. Body height was measured to the nearest 0.5 cm. Body mass index (BMI) was calculated as the body weight in kilograms divided by the body height in meters squared. Venous blood samples were drawn after overnight fasting for measurement of serum triglycerides, serum total, high-density lipoprotein (HDL) and low-density lipoprotein (LDL) cholesterol, and serum creatinine and uric acid, and plasma glucose.

Diabetes mellitus was defined as a plasma glucose concentration ≥7.0 mmol/l, or use of antidiabetic drugs, or as a diagnosis self-reported or documented in practice or hospital records. Dyslipidemia included hypertriglyceridemia defined as a serum triglycerides concentration ≥2.30 mmol/l, hypercholesterolemia defined as a serum total cholesterol concentration ≥6.20 mmol/l or a serum LDL cholesterol concentration ≥4.10 mmol/l, a serum HDL cholesterol concentration <1.00 mmol/l, or the current use of lipid-lowering medications, or as a diagnosis self-reported or documented in practice or hospital records.^17^ Cardiovascular diseases refer to past medical history of cardiac, cerebrovascular, and peripheral arterial diseases according to the International Classification of Diseases, 9^th^ Revision (ICD-9, codes 391.001 to 448.901) or International Classification of Diseases, 10^th^ Revision (ICD-10, codes I01.001 to I98.801). The diagnosis of cardiovascular diseases was either documented in the practice or hospital medical records, self-reported, or based on the use of cardiovascular therapeutic agents.

### Statistical Analysis

The SAS software version 9.4 (SAS Institute Inc., Cary, NC, USA) was used for data management and statistical analysis. Means and proportions were compared using the student’s *t*-test and Fisher’s exact test, respectively. The morning-to-evening changes in blood pressure and pulse rate were calculated by subtracting the values in the morning from those in the evening. Negative values indicate a reduction from morning to evening. We performed univariate analysis and multiple stepwise linear regression analysis to investigate the determinants of the morning-to-evening changes in home blood pressure and pulse rate. In the multiple regression models, we forced age and sex and considered BMI, current smoking and alcohol intake, hypertension duration, history of cardiovascular disease, diabetes mellitus and dyslipidemia, serum triglycerides, serum total, HDL and LDL cholesterol, plasma fasting glucose, serum creatinine and uric acid and the use of angiotensin-converting enzyme inhibitor (ACEIs), angiotensin receptor blockers (ARBs), α-blockers, β-blockers, and calcium channel blockers (CCBs) as covariates to enter and stay at a significance level set at 0.05. Furthermore, we performed analyses of covariance according to the sex-specific quartile distributions to investigate the interrelationship between the morning-to-evening changes in home blood pressure and pulse rate, while accounting for sex, age and the determinants identified in the above multiple regression analyses. Finally, we performed analyses on the control of office blood pressure (<140/90 mm Hg) in relation to the morning-to-evening changes in home pulse rate at baseline. Double product was calculated by systolic blood pressure times pulse rate. *P* values < 0.05 were considered statistically significant.

## RESULTS

### Characteristics of the Study Participants

The 4,787 participants had a mean (±SD) age of 66.1 (±7.5) years and included 2,366 (49.4%) men. Men and women differed significantly in almost all characteristics (*P* ≤ 0.030, **Table 1**), except for age, serum triglycerides, and history of dyslipidemia. Men, compared with women, had a significantly higher office (133.1/83.4 vs. 130.2/80.0 mm/Hg) and home systolic/diastolic blood pressure (129.4/81.2 vs. 127.0/78.1 mm/Hg).

**Table 1.** Characteristics of the study participants by gender.

Characteristic	Men	Women	*P* value
	(*n* = 2,366)	(*n* = 2,421)	
Age, years	65.9 ± 7.3	66.3 ± 7.7	0.067
Body mass index, kg/m^2^	25.4 ± 3.0	24.9 ± 3.3	<0.001
Current smoking, *n* (%)	743 (31.4)	15 (0.6)	<0.001
Alcohol intake, *n* (%)	793 (33.5)	20 (0.8)	<0.001
Hypertension duration, years	11.9 ± 9.4	10.5 ± 9.4	<0.001
Number of antihypertensive medications, *n*	1.7 ± 0.9	1.5 ± 0.8	<0.001
Office blood pressure measurement			
Systolic blood pressure, mmHg	133.1 ± 17.1	130.2 ± 17.3	<0.001
Diastolic blood pressure, mmHg	83.4 ± 10.6	80.0 ± 10.5	<0.001
Pulse rate, beats/min	75.0 ± 11.0	74.4 ± 10.2	0.029
Home blood pressure measurement			
Morning systolic blood pressure, mmHg	130.3 ± 13.6	127.9 ± 13.7	<0.001
Morning diastolic blood pressure, mmHg	82.3 ± 8.8	78.9 ± 8.6	<0.001
Morning pulse rate, beats/min	70.8 ± 9.5	70.3 ± 8.6	0.025
Evening systolic blood pressure, mmHg	128.4 ± 13.3	126.0 ± 13.2	<0.001
Evening diastolic blood pressure, mmHg	80.2 ± 8.3	77.4 ± 8.4	<0.001
Evening pulse rate, beats/min	74.6 ± 9.7	72.8 ± 8.6	<0.001
Double product (mmHg·beats/min)			
Office	9,975 ± 1,919	9,666 ± 1,772	<0.001
Home morning	9,216 ± 1,497	8,973 ± 1,386	<0.001
Home evening	9,571 ± 1,551	9,164 ± 1,418	<0.001
Blood biochemistry			
Plasma fasting glucose, mmol/L	6.1 ± 1.9	6.0 ± 1.8	0.030
Serum total cholesterol, mmol/L	4.5 ± 1.1	5.0 ± 1.2	<0.001
Serum triglycerides, mmol/L	1.7 ± 1.1	1.7 ± 1.1	0.238
Serum high-density lipoprotein cholesterol, mmol/L	1.3 ± 0.4	1.4 ± 0.5	<0.001
Serum low-density lipoprotein cholesterol, mmol/L	2.6 ± 0.9	2.8 ± 0.9	<0.001
Serum creatinine, μmol/L	82.3 ± 35.9	65.7 ± 27.0	<0.001
Serum uric acid, μmol/L	355.3 ± 93.2	298.6 ± 82.8	<0.001
Diabetes mellitus, *n* (%)	733 (31.0)	637 (26.3)	0.001
Dyslipidemia, *n* (%)	1,340 (56.6)	1,353 (55.9)	0.620
Cardiovascular disease, *n* (%)	518 (21.9)	403 (16.6)	<0.001

Values are mean ± standard deviation, or number of participants (% of column total).

The median (interquartile range) number of days and readings of home blood pressure measurement was 7 (5–7) and 42 (39–48), respectively. The measurement time was between 06:00 and 08:00 and between 18:00 and 21:00 in the morning and evening, respectively (**Supplementary Table 1****).**

**Figure 1.**
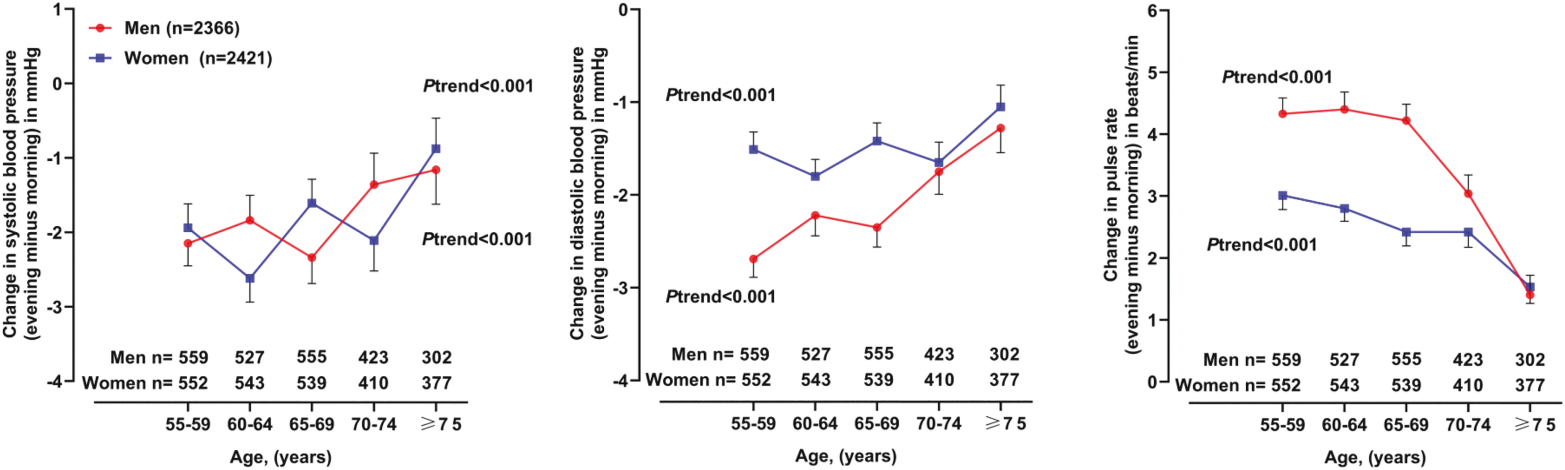
Morning-to-evening changes in home blood pressure and pulse rate by sex and age group. Symbols represent the mean value. Vertical lines denote standard error. The number of patients is given for each age subgroup at the bottom of the figure in men and women separately. The *P* value for trend is also given for men and women separately.

Home systolic/diastolic blood pressure decreased from 129.0/80.6 mmHg in the morning to 127.2/78.7 mmHg in the evening by a mean change of −1.9 ± 7.8/−1.8 ± 4.7 mmHg. Home pulse rate increased from 70.5 beats/min in the morning to 73.7 beats/min in the evening by a mean change of +3.1 ± 5.8 beats/min. Home double product increased from 9,093 ± 1,447 in the morning to 9,365 ± 1,499 in the evening by a mean change of 272 ± 832. The morning-to-evening changes in blood pressure tended to be smaller with age advancing in both sexes (*P*trend < 0.001, **Figure 1**).

### Determinants of the Morning-to-Evening Changes in Home Blood Pressure and Pulse Rate

In univariate analysis, the morning-to-evening changes in blood pressure and pulse rate were associated with several demographic and lifestyle factors and anthropometric and biochemical measurements (**Supplementary Table 2**). In multiple stepwise linear regression, the morning-to-evening changes in home blood pressure and pulse rate remained associated with most of the determinants identified in the above univariate analysis (Table 2).

**Table 2.** Multiple stepwise linear regression analysis on the morning-to-evening change in blood pressure and pulse rate (*n* = 4,787).

Variable	Change in SBP, mmHg		Change in DBP, mmHg		Change in pulse rate, beats/min	
	β ± SE	*P* value	β ± SE	*P* value	β ± SE	*P* value
Model						
Age, + 5 years	0.23 ± 0.08	0.003	0.16 ± 0.05	<0.001	−0.21 ± 0.06	<0.001
Male sex	0.28 ± 0.26	0.279	−0.22 ± 0.16	0.171	0.39 ± 0.19	0.038
Body mass index, kg/m^2^	-	-	−0.05 ± 0.02	0.029	0.21 ± 0.03	<0.001
Current smoking (yes vs. no)	1.93 ± 0.35	<0.001	0.80 ± 0.21	<0.001	1.25 ± 0.25	<0.001
Alcohol intake (yes vs. no)	−2.11 ± 0.34	<0.001	−1.52 ± 0.21	<0.001	1.30 ± 0.25	<0.001
Hypertension duration, + 5 years	−0.17 ± 0.06	0.007	−0.08 ± 0.04	0.047	−0.15 ± 0.05	0.001
Serum HDL cholesterol, + 0.1 mmol/L	-	-	0.38 ± 0.15	0.013	-	-
Serum triglycerides, + 0.1 mmol/L	-	-	-	-	0.21 ± 0.07	0.004
ACEIs use (yes vs. no)	−1.39 ± 0.49	0.004	−0.78 ± 0.29	0.007	-	-
ARBs use (yes vs. no)	−1.11 ± 0.26	<0.001	−0.77 ± 0.16	<0.001	0.60 ± 0.16	<0.001
α-blockers use (yes vs. no)	-	-	-	-	−2.87 ± 0.66	<0.001
β-blockers use (yes vs. no)	-	-	−0.57 ± 0.18	0.002	−0.62 ± 0.22	0.005
CCBs use (yes vs. no)	−0.57 ± 0.27	0.033	−0.41 ± 0.16	0.011	-	-

^*^The *R*² statistic for the final linear regression model was 0.02, 0.03, and 0.06 for the morning-to-evening changes in systolic (SBP) and diastolic blood pressure (DBP) and pulse rate, respectively.

In a linear regression model, we forced age and sex, and considered body mass index, current smoking and alcohol intake, hypertension duration, history of cardiovascular disease, diabetes mellitus and dyslipidemia, serum triglycerides, serum total, high-density lipoprotein (HDL) and low-density lipoprotein cholesterol, plasma fasting glucose, serum creatinine and uric acid, and the use of angiotensin-converting enzyme inhibitors (ACEIs), angiotensin receptor blockers (ARBs), α-blockers, β-blockers, and calcium channel blockers (CCBs) as covariates for entry and stay at a significance of *P* ≤ 0.05.

Further adding pulse rate in the model for blood pressure did not materially change the associations for the above-identified determinants. However, further adding blood pressure in the model for pulse rate changed the association with male sex, although the associations for all the other above-identified determinants remained unchanged.

### Interrelationship Between the Morning-to-Evening Changes in Home Blood Pressure and Pulse Rate

**Figure 2.**
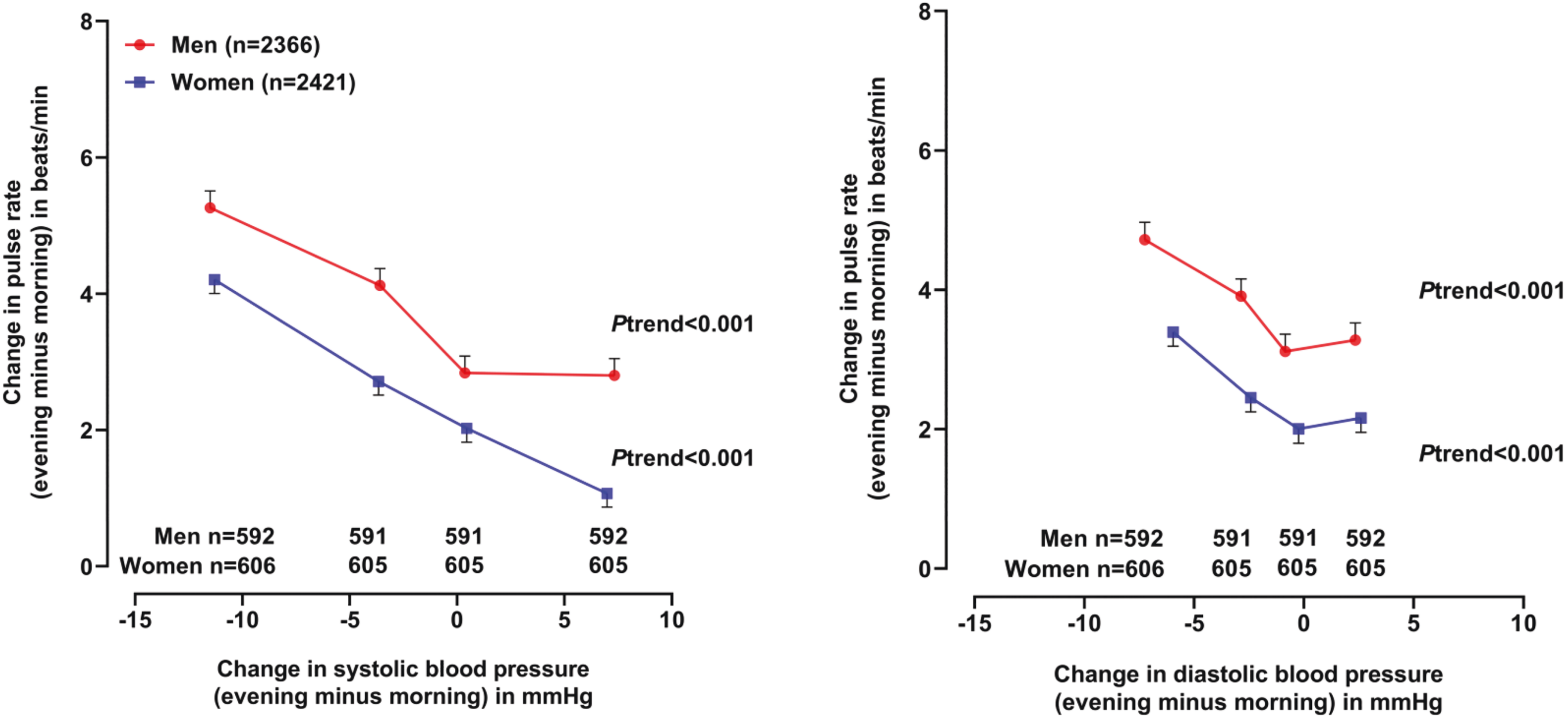
Relationship between the morning-to-evening changes in home pulse rate and systolic (left panel) and diastolic blood pressure (right panel) by sex. Symbols represent mean value adjusted for age, body mass index, current smoking and alcohol intake, hypertension duration, serum low-density lipoprotein cholesterol, angiotensin receptor blocker use, α-blocker use, and β-blocker use. Vertical lines denote standard error. The *P* values for trend are given.

The morning-to-evening changes in home pulse rate were inversely associated with those in home systolic and diastolic blood pressure (*r* = −0.20 and −0.12, respectively, **Figure 2**). In the presence of a morning-to-evening decrease in systolic blood pressure, there was an increase in home pulse rate (+5.2 beats/min in men and +4.1 beats/min in women). In the presence of no change or an increase in blood pressure, there was still a smaller increase in home pulse rate in both men (+2.8 beats/min) and women (+1.0 beats/min). After adjustment for the above-identified factors for the morning-to-evening changes in pulse rate, the results were confirmatory.

### Relationship Between Morning-to-Evening Changes in Home Pulse Rate and Office Blood Pressure Control

**Figure 3.**
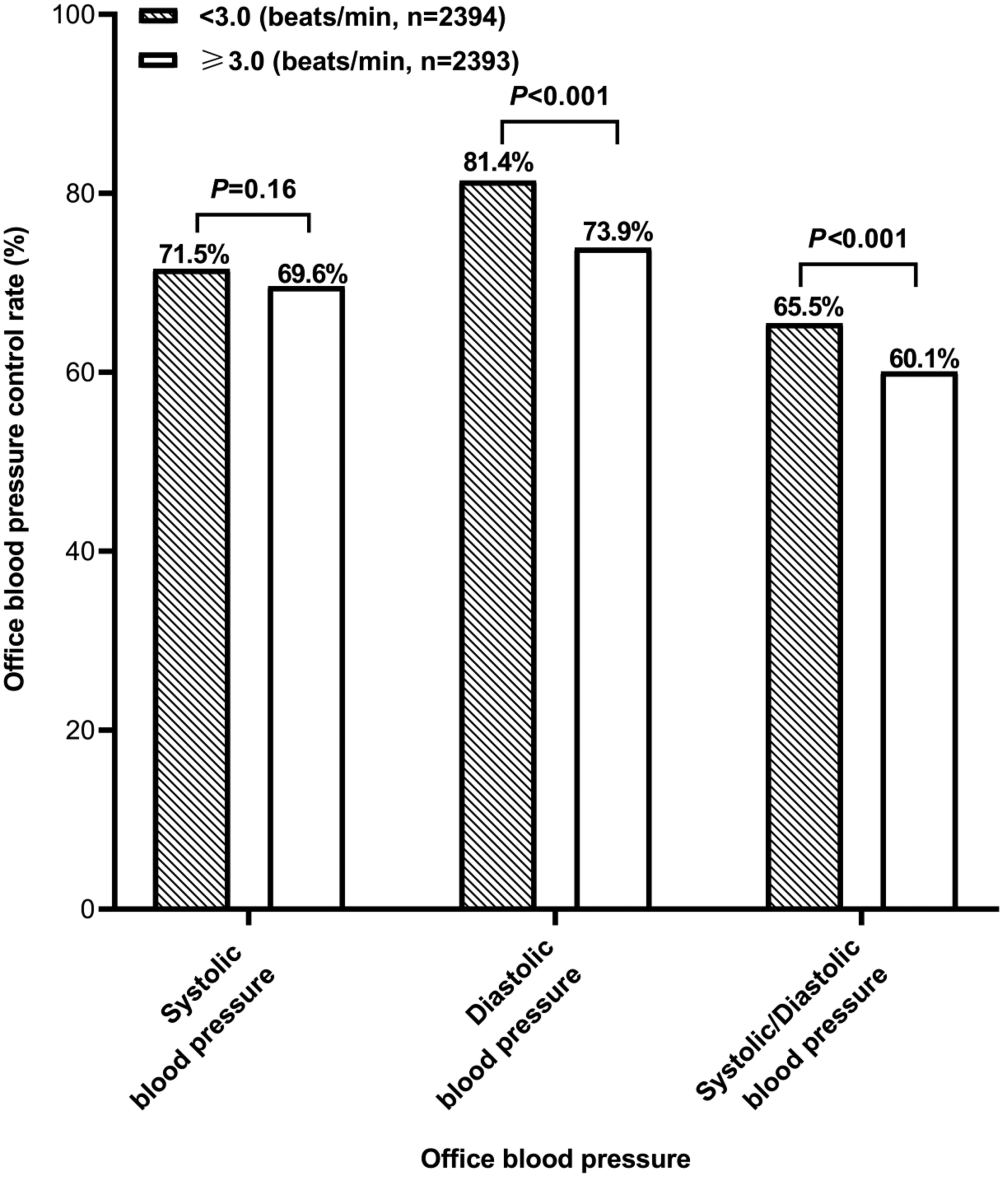
Office blood pressure control rate at baseline in patients below (filled bar) or above (open bar) the median of the morning-to-evening changes in home pulse rate. The percentage is given along the bars. The *P* value is also given.

At baseline, the control rate was lower for office diastolic blood pressure alone (73.9% vs. 81.4%, *P* < 0.001) and combined office systolic and diastolic blood pressure (60.1% vs. 65.5%, *P*=<0.001), but not office systolic blood pressure alone (69.6% vs. 71.5%, *P* = 0.16), in patients with a morning-to-evening change in pulse rate above the median (≥3.0 beats/min) than below the median (<3.0 beats/min, **Figure 3**).

## DISCUSSION

The key finding of our study was that there were interrelated morning-to-evening changes in home blood pressure and pulse rate, being on average a drop and rise, respectively. The size of these changes was associated with several demographic and lifestyle factors, and anthropometric and biochemical measurements, and the use of various antihypertensive drugs. In addition, patients with a greater increase in home pulse rate had a poor control status of office blood pressure.

Our observation on the lower blood pressure in the evening than in the morning is in line with the results of several previous studies in Asia^11,12,18–23^ but not those in Europe.^24,25^ Several large-scale studies in Asia, mainly Japan, reported the morning and evening home blood pressure and pulse rate values.^11,12,18–22,26^ In a recent study involving five Japanese general populations (*n* = 9,801), the mean systolic/diastolic blood pressures were 129.6/77.0 mmHg in the morning and 122.1/70.5 mmHg in the evening by a mean change of −7.5/−6.5 mmHg.^26^ In 3,400 treated patients enrolled in the J-HOME (The Japan Home versus Office Blood Pressure Measurement Evaluation) study, home morning and evening systolic/diastolic blood pressure changed from 139.6/81.7 mmHg in the morning to 133.7/76.9 mmHg in the evening by a mean change of −5.9/−4.8 mmHg.^19^ In 21,569 treated hypertensive patients enrolled in the HONEST (Home BP measurement with Olmesartan Naive patients to Establish Standard Target blood pressure) study, the corresponding values were 151.6/87.1, 150.1/86.6, and −1.5/−0.5 mmHg, respectively.^27^ Several large-scale studies in Europe also reported the morning and evening blood pressure and pulse rate values. In 1919, participants in the Finn-Home general population study, home systolic/diastolic blood pressure changed from 128.1/80.2 mmHg in the morning to 131.1/80.2 mmHg in the evening by a mean change of 3.0/0.0 mmHg.^24^ In 233 untreated hypertensive patients also from Finland, the corresponding values were 137.1/92.4, 140.8/93.4, and 3.7/1.0 mmHg, respectively. It is well-agreed that the measurement time after or before super has played a major role in the differentiation between the two ethnicities in the blood pressure changes from morning to evening.

Our findings on the morning-to-evening changes in home pulse rate may have straightforward explanations. The increase in pulse rate from morning to evening is rather a consequence than a cause of the decrease in blood pressure. Increasing pulse rate probably behaves as a compensating factor for the morning-to-evening drop in blood pressure, directly via increasing cardiac output^28^ and indirectly via various regulatory mechanisms associated with increasing pulse rate, such as the activation of sympathetic nervous system and renin-angiotensin-aldosterone system.^29^ In our present study, there was a possible morning-to evening increase in cardiac output as indicated by the observed slight increase in the double product that was calculated by systolic blood pressure times pulse rate, and can reflect cardiac output.^30,31^ In addition, our study participants were treated with various antihypertensive drugs, which may not only reduce blood pressure but also potentially influence the changes in blood pressure and pulse rate during day, especially β-blockers and ARBs.

The observation that the morning-to-evening changes in both blood pressure and pulse rate were associated with several demographic and lifestyle factors and anthropometric and biochemical measurements suggests that these changes might be related to physical activity, organ damage, decay of the regulatory system, use of various antihypertensive drugs, and so on.^32,33^ For instance, the smaller drop in blood pressure and rise in heart rate associated with older age might be attributable to decreased physical activity. For most of the patients, antihypertensive drugs were taken in the morning, and morning blood pressure and heart rate tended to increase before drug intake. The greater drop in blood pressure and rise in heart rate in patients with a greater BMI and in alcohol drinkers might be attributable to dysregulation of the nervous and hormonal systems.^34–37^ In addition, the morning-to-evening changes in systolic and diastolic blood pressure had similar determinants except that diastolic but not systolic changes were associated with BMI and serum HDL cholesterol. This finding is not entirely understood. It is possible that diastolic blood pressure, which is more reflective of peripheral vascular resistance and less influenced by arterial stiffness than systolic blood pressure,^38,39^ may be more sensitive to metabolic alterations, such as obesity and dyslipidemia.^40,41^

Our new finding on the poor blood pressure control in those patients with a greater increase in home pulse rate from morning to evening is not entirely understood. Because systolic blood pressure is less influenced by pulse rate than diastolic blood pressure, our observation on the stronger association of the morning-to-evening changes in pulse rate with diastolic than systolic blood pressure control might be pathophysiologically relevant.^15,42^ The greater increase in pulse rate from morning to evening, possibly induced by the blood pressure drop, might indicate severer organ damage and cardiovascular dysregulation. Nonetheless, in the Ohasama study, pulse rate in the morning (hazard ratio 1.17, 95% CI: 1.05–1.30) and evening (hazard ratio 1.16, 95% CI: 1.04–1.29) was similarly associated with the risk of cardiovascular mortality.^43^ In addition, in a recent analysis of the Hypertension Objective treatment based on Measurement by Electrical Devices of Blood Pressure (HOMED-BP) study, home pulse rate was superior to office pulse rate as a predictor of all-cause mortality, whether before (hazard ratio 1.52, 95% CI: 1.24–1.92) or after antihypertensive drug treatment (hazard ratio 1.70, 95% CI: 1.70–2.08).^44^ Taken together these previous results, our finding and recent Chinese^45^ and European^46^ guideline recommendations on faster pulse rate as a predictor or risk factor, there is a need of more research on home pulse rate in treated hypertension.

Our study should be interpreted within the context of its strengths and limitations. The same automated blood pressure measuring device was used for the office and home blood pressure measurements. The device difference was therefore minimal. Blood pressure was automatically transmitted to a digital platform. However, our registry was cross-sectional. Second, although a standardized protocol was applied for blood pressure measurement, there was still possible divergence in data collection between hospitals and community health centers. Third, we did not perform autonomic nervous system assessments, such as heart rate variability, which limits our ability to characterize the potential mechanisms underlying the observed compensatory responses to changes in blood pressure.

In conclusion, our cross-sectional study showed that treated Chinese patients with hypertension had interrelated morning-to-evening changes in home blood pressure and pulse rate, being on average a drop and rise, respectively. These changes might be clinically relevant in blood pressure control, and probably in cardiovascular prevention as well.

## Supplementary Material

hpaf137_suppl_Supplementary_Materials_1

## Data Availability

Data described in the manuscript, code book, and analytic code will be made available upon reasonable request.
