# Supplementary material for: Interrelationship Between the Morning-to-Evening Changes in Home Blood Pressure and Pulse Rate
Source: Am J Hypertens. 2025 Jul 20;38(12):1051–9. doi: 10.1093/ajh/hpaf137 (PMC12620026; doi:10.1093/ajh/hpaf137)
Supplement: hpaf137_suppl_Supplementary_Materials_1 [file hpaf137_suppl_supplementary_materials_1.docx]

**Interrelationship between the morning-to-evening changes in home blood pressure and pulse rate**

Short title: Morning-to-evening changes in home BP and PR

**Authors:** Jia-Bo Zhu^a^, Qian-Hui Guo^a^, Yi Zhou^a^, Wen-Yuan-Yue Wang^b^, Yuan-Yuan Kang^a^, Xiao-Fei Ye^a^, Xin-Yu Wang^a^, Ming-Xuan Li^a^, Yan Li^a^, Ji-Guang Wang^a,b^

**Affiliations**

^a^ Department of Cardiovascular Medicine, Centre for Epidemiological Studies and Clinical Trials, Shanghai Key Laboratory of Hypertension, The Shanghai Institute of Hypertension, National Research Centre for Translational Medicine at Shanghai, Ruijin Hospital, Shanghai Jiao Tong University School of Medicine, Shanghai, China

^b^ School of Public Health, Shanghai Jiao Tong University School of Medicine, Shanghai, China.

**Correspondence to** Ji-Guang Wang, MD, PhD, The Shanghai Institute of Hypertension, Ruijin 2nd Road 197, Shanghai 200025, China. Phone: +86-21-64370045-610911; Fax: +86-21-64662193; Email: [jiguangwang@rjh.com.cn](mailto:jiguangwang@rjh.com.cn)

**Supplementary Table 1.** Quality control of home blood pressure monitoring (n=4787).

| Home Blood pressure monitoring | Number of measurement days or blood pressure readings per patient | | | |
| --- | --- | --- | --- | --- |
|  | Median | P25-P75 | P10-P90 | P5-P95 |
| Number of measurement days | 7 | 5 to 7 | 7 to 8 | 4 to 9 |
| Measurement time |  |  |  |  |
| Morning | 7 | 6 to 8 | 5 to 9 | 5 to 10 |
| Evening | 20 | 18 to 21 | 17 to 22 | 16 to 22 |
| Number of blood pressure readings | | | | |
| Morning and evening | 42 | 39 to 48 | 39 to 60 | 30 to 75 |
| Morning | 21 | 18 to 24 | 17 to 30 | 15 to 31 |
| Evening | 21 | 21 to 24 | 18 to 21 | 15 to 21 |

P25-P75 indicates the 25th-75th percentile interval, P10-P90 the 10th-90th percentile interval, P5-P95 the 5th-95th percentile interval.

.

**Supplementary Table 2.** Univariate linear regression analysis on the morning-to-evening blood pressure and heart rate (n=4787).

| Variable | Change in systolic blood pressure | |  | Change in diastolic blood pressure | |  | Change in heart rate | |
| --- | --- | --- | --- | --- | --- | --- | --- | --- |
|  | r | *P* value |  | r | *P* value |  | r | *P* value |
| Age, +5 years | 0.040 | 0.006 |  | 0.058 | <0.001 |  | -0.111 | <0.001 |
| Male sex | 0.002 | 0.912 |  | -0.069 | <0.001 |  | 0.109 | <0.001 |
| Body mass index, kg/m^2^ | -0.046 | 0.001 |  | -0.062 | <0.001 |  | 0.138 | <0.001 |
| Current smoking (yes *vs.* no) | 0.050 | <0.001 |  | -0.007 | 0.620 |  | 0.136 | <0.001 |
| Alcohol intake (yes *vs.* no) | -0.068 | <0.001 |  | -0.120 | <0.001 |  | 0.148 | <0.001 |
| Hypertension duration, +5 years | -0.042 | 0.003 |  | -0.046 | 0.002 |  | -0.051 | <0.001 |
| Plasma fasting glucose, mmol/L | 0.015 | 0.301 |  | -0.002 | 0.913 |  | 0.025 | 0.082 |
| Serum total cholesterol, mmol/L | 0.008 | 0.583 |  | 0.023 | 0.115 |  | 0.017 | 0.245 |
| Serum triglycerides, mmol/L | -0.002 | 0.864 |  | -0.014 | 0.347 |  | 0.067 | <0.001 |
| Serum high-density lipoprotein cholesterol, mmol/L | 0.008 | 0.564 |  | 0.049 | 0.001 |  | -0.018 | 0.220 |
| Serum low-density lipoprotein cholesterol, mmol/L | 0.023 | 0.114 |  | 0.029 | 0.042 |  | 0.012 | 0.419 |
| Serum creatinine, μmol/L | 0.025 | 0.085 |  | -0.004 | 0.782 |  | 0.001 | 0.937 |
| Serum uric acid, μmol/L | -0.022 | 0.126 |  | -0.047 | 0.001 |  | 0.067 | <0.001 |
| History of diabetes mellitus (yes *vs*. no) | 0.015 | 0.304 |  | -0.005 | 0.731 |  | 0.016 | 0.281 |
| History of dyslipidemia (yes *vs*. no) | -0.015 | 0.309 |  | -0.021 | 0.141 |  | 0.040 | 0.005 |
| History of cardiovascular disease (yes *vs*. no) | -0.022 | 0.130 |  | -0.025 | 0.089 |  | -0.014 | 0.327 |
| CCB use (yes *vs*. no) | -0.007 | 0.635 |  | -0.011 | 0.442 |  | -0.025 | 0.086 |
| ACEI use (yes *vs*. no) | -0.023 | 0.107 |  | -0.020 | 0.168 |  | 0.012 | 0.399 |
| ARB use (yes *vs*. no) | -0.054 | <0.001 |  | -0.071 | <0.001 |  | 0.055 | <0.001 |
| α-blocker use (yes *vs*. no) | 0.020 | 0.171 |  | -0.011 | 0.440 |  | -0.055 | <0.001 |
| β-blocker use (yes *vs*. no) | -0.027 | 0.063 |  | -0.049 | <0.001 |  | -0.046 | 0.001 |

ARB, indicates angiotensin receptor blocker; ACEI, angiotensin-converting enzyme inhibitor; CCB, calcium channel blocker.
